# Consumption of Raw Orange, 100% Fresh Orange Juice, and Nectar- Sweetened Orange Juice—Effects on Blood Glucose and Insulin Levels on Healthy Subjects

**DOI:** 10.3390/nu11092171

**Published:** 2019-09-10

**Authors:** Dimitrios Papandreou, Emmanouella Magriplis, Myriam Abboud, Zainab Taha, Eleftheria Karavolia, Christos Karavolias, Antonis Zampelas

**Affiliations:** 1 Department of Health Sciences, CNHS, Zayed University, Abu Dhabi 144534, UAE; Myriam.abboud@zu.ac.ae (M.A.); Zainab.taha@zu.ac.ae (Z.T.); 2 Department of Food Science and Human Nutrition, Agricultural University of Athens, 11855 Athens, Greece; emagriplis@eatsmart.gr (E.M.); azampelas@aua.gr (A.Z.); 3 Faculty of Medicine, University of Groningen, 9712KB Groningen, The Netherlands; elka19@hotmail.com; 4 Doctor’s Medical Center, Abu Dhabi, Abu Dhabi 46400, UAE; karavolias@hotmail.com

**Keywords:** raw orange, orange juice, glucose, insulin, Emirati, diabetes type 2

## Abstract

Objective: The aim of this study is to investigate the effect of consumption of raw orange (RO), 100% fresh orange juice (FOJ), and nectar-sweetened orange juice (NSOJ) on postprandial glucose and insulin levels in non-diabetic young Emirati women. Research Methods: This is a prospective, three-way, crossover study design. Blood records of thirteen normal weight and seven healthy obese university students were analyzed from Zayed University on three random days with the following three meal samples: 2 ROs, 100% FOJ, and NSOJ. Venous blood was collected at 0, 30, 60, 90, and 120 minutes after the respective meal consumption. Statistical analyses included repeated measures analysis of variance and calculations of the area under the glucose and insulin curves (AUC) for each one of the meal samples. Results: Total fasting glucose and insulin levels did not differ by treatment in the normal versus obese group. All three meals had no significant effects on the plasma glucose levels. However, there was a significant change in plasma insulin concentrations at 120 min compared with that at 0 min for RO: −14 (−27.05, −0.90, *P* < 0.001); 100% FOJ −13.7 (−28.80, 1.44, *P* < 0.001); and NSOJ: −9.2 (−28.75, 10.30, *P* < 0.001). Conclusions: This study shows that whole fresh fruit, 100% fruit juice, and sweetened fruit juice did not have a significant effect on the blood glucose levels in non-diabetic Emirati university students. However, a significant decrease in insulin response and HOMA-IR on all three sample meals was observed.

## 1. Introduction

Type 2 diabetes mellitus (T2DM) is one of the main causes of mortality and morbidity worldwide. In 2012, the International Diabetes Federation (IDF) reported that 371 million people have diabetes and that this number will is projected to increase to 552 million by 2030 [1]. The major complications of T2DM is its significant impact on the quality of life, thereby posing a serious threat to community [2]. Thus, it is vital to identify the modifiable risk factors that would eventually lead to attenuate the incidence of this major health issue.

The numbers for patients with T2DM in Arab countries are quite alarming. The Middle East has the second highest rate of increase in diabetes in the world, and this is expected to reach 96% in 2035 if no action is taken [3]. The major factors that seem to be associated with T2DM in Arab people, are genetics, obesity, urbanization, sedentary lifestyles, and high-calorie food consumption (diet rich in fats and sugars and low in fibers) [4].

Many studies have shown that patients with diabetes have two–four times higher risk of developing cardiovascular diseases (CVDs). Therefore, one of the strategies to reduce the major incidence of CVD in patients with T2DM is to control their high blood sugar levels [5]. The control of postprandial glucose and insulin levels is critical not only for patients with diabetes but also for healthy people in order to prevent glucose intolerance and insulin resistance cycling response [6].

An increasing number of studies suggests that eating healthy especially adequate consumption of high fiber and phytochemical-rich fruits and vegetables could delay hyperglycemia and T2DM [7,8]. A recent systematic review by Xi and his colleagues incorporating 191,686 participants has shown that limiting the consumption of sugar-sweetened beverages (SSBs) may prevent progression of T2DM [9]. Consuming SSBs increases the total calorie intake, leading to unhealthy weight gain and increased risk of developing CVD. However, whether or not the intake of fruit juices contributes to T2DM development remains unclear [10,11]. 

Fruit juices (FJs) have excess calories that may be attributed to the addition of sugar [12]. Many prospective studies have demonstrated that fruit fiber is not significantly related to the low risk of T2DM [13,14]. Furthermore, two recent systematic reviews by Wang et al. [15] and Murphy et al. [16] have shown that 100% FJs may exert a neutral effect on glycemic and insulin control. 

This study aimed to investigate the effect of consuming raw orange (RO), 100% fresh orange juice (FOJ), and nectar-sweetened orange juice (NSOJ) on postprandial glucose and insulin levels in non-diabetic Emirati women and to compare the difference of those response overtime.

## 2. Methods

Sixty-two healthy female Emirati students were initially assessed to participate in the study from a sample of 500 students. Twenty-two patients were excluded from the study. Participants included in this study were female, aged 20–22 years with no medical problems. The exclusion criteria were medical issues such as diabetes, cardiovascular/liver/kidney disease, taking any type of medication or supplements, or being on a specific diet. Finally, thirteen normal-weight and seven obese females aged 20–22 years old participated in the study. The sample size was considered adequate to achieve 90% statistical power for detecting a difference of 15mg/dL of blood glucose levels in the 2-h test. Specifically, the power was calculated for a = 0.05; effect size d = 0.3; three groups cross-over design, F statistic between and within group analysis for 5 repeated measurements, non-sphericity correction (ε) = 1.0. This study obtained ethical approval from the Research Ethics Committee at Zayed University (ZU_081_F) and the Doctor’s Medical Center (TDMC_25_2015). All subjects provided written informed consent prior to participation. The study was retrospectively registered with number: ISRCTN10834747.

### 2.1. Intervention

In this prospective crossover study, each subject underwent the test on all three intervention tests. The order that the intervention tests were given to all participants was randomized (1st, 2nd and 3rd sample was randomly selected, 3 days apart to decrease possible outcome effects). During this process the first sample to be given was the 2 oranges, the second intervention was 100% fruit juice and lastly the sweetened orange juice). Participants were not randomized to the interventions; they all received the samples as selected on the same day (Table 1). The effects of the three sample fruit meals were evaluated by measuring the glucose and insulin responses until 2-h post-prandially. NSOJ was obtained from the supermarket. Plasma blood glucose and insulin levels were measured at 0, 30, 60, 90, and 120 min T0, T30, T60, T90 and T120), respectively. A 24-h diet recall was performed one day before the tests on all three occasions out to ensure that there no major changes in the dietary intake of the participant

### 2.2. Anthropometric Data

Body weight and heights of the fasting patients were measured on a digital scale whilst participants were wearing light indoor clothing (SECA 600, Germany). Obesity was calculated based on the International Obesity Task Force (IOTF) criteria [17]. A 24-h dietary recall was performed the day before the procedure by a registered dietitian, and data were analyzed using the Nutritionist IV software (USA).

### 2.3. Blood Collection

Plasma glucose and insulin levels were measured in the morning after fasting overnight. Blood samples were drawn by a certified nurse by venipuncture into 10-mL empty evacuated and placed on cold tubes. The tubes were immediately centrifuged at 2000× *g* for 10 min. Plasma glucose levels were measured using a hexokinase enzymatic reference method (Cobas, Roche USA). Fasting insulin levels were measured using the electrochemiluminescence method (Cobas 6000 analyzer, Roche, USA). Insulin resistance model was calculated as follows:

HOMA-IR = [FI (μU/mL) × FG (mmol/L)/22.5]

### 2.4. Statistics

Each individual’s baseline data was considered served as their own control, thereby removing the effect of between-subject variation. Demographic data were presented by weight status because excess weight may affect the postprandial test. This was accounted for in the analysis, as well as age, total energy, and fiber intake to avoid incorporating error in the results. The results and analyses of metabolic outcomes were not presented by weight status because if stratification were performed in this small sample population, it would decrease the power of the study. However, this data is presentined in Supplementary Materials Table S1 and Table S2. The positive incremental area under the curve (iAUC) was calculated, which is defined as the area above the baseline value (fasting); hence, only the positive area under the curve (AUC) values were summed, according to Wolever et al. [18]. In case that the net incremental area was estimated, then AUC is the area above the baseline; however, in this case, negative values were considered by being deducted. 

Normality was examined using k-density plots in association with the Kolmogorov-Smirnov test. For skewed distribution, data were log transformed for the final analysis. Mean differences were examined using Student’s T-test. One-way analysis of variance (ANOVA) was performed multiple variables when normally distributed and Wilcoxon rank sum (Mann-Whitney) test was performed for skewed variables. Mean energy (kcal/day) and macronutrient intake (gr/day) were compared using ANOVA at each group/treatment level to examine between-group variation. In order to consider for within-subject variation, mixed effects regression (mixed and xt: mixed) for repeated measures was used to (i) evaluate the differences between the three samples tested at each time point of the postprandial test (T0, T30, T60, T90 and 120) defined as the treatment effect, (ii) evaluate the differences observed within each group, defined as the time effect, and (iii) evaluate the treatment x time interaction effect. 

Adjustments were made for body mass index (BMI), energy (kcal), and fiber (grams) intake, for each treatment group. The time to peak and the positive iAUC for insulin and glucose was the between-group (sample) factors, and the mean glucose and insulin measurements at 0, 30, 60, 90 and 120 m (of the postprandial test) were the within-group factors. Two-tailed P-values were reported, and statistical significance was set at the level of 0.05. All analyses were conducted using STATA 14.0 (StataCorp LLC, TX, USA).

## 3. Results

Obese subjects had significantly higher BMI, greater calorie intake, and lower fiber consumption than the normal weight subjects (*P* < 0.001). No statistically significant changes were observed for blood glucose (*P* = 0.789) and insulin (*P* = 0.763) levels in both groups. All baseline characteristics are presented in Table 2. 

Glucose: The mean changes in the plasma blood glucose levels had similar responses at all of the time intervals (*P* = 0.462) after all three sample meals. In addition, there was no significant change among the groups (Table 3).

Plasma Insulin: The mean changes in the plasma insulin concentrations (T120-T0) were significantly lowered after the consumption of RO [−14 (−27.05, −0.90, *P* < 0.001)], FOJ [−13.7 (−28.8, 1.44, *P* < 0.001)], and NSOJ [−9.2 (−28.75, 10.30, *P* < 0.001)]. However, there was no significant change in the comparisons among the groups (Table 3).

HOMA-IR: The mean changes in HOMA-IR (T120-T0) were observed to be significantly lowered in RO [−0.3 (−0.61, −0.03, *P* < 0.001)], FOJ [−0.3 (−0.67, 0.01, *P* < 0.001)], and NSOJ [−0.2 (−0.66, 0.22, *P* < 0.001)] (Table 3). 

**Figure 1:**
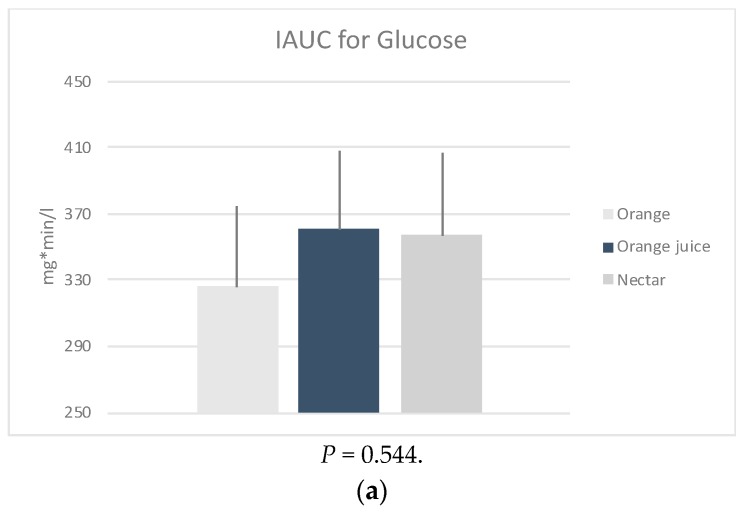

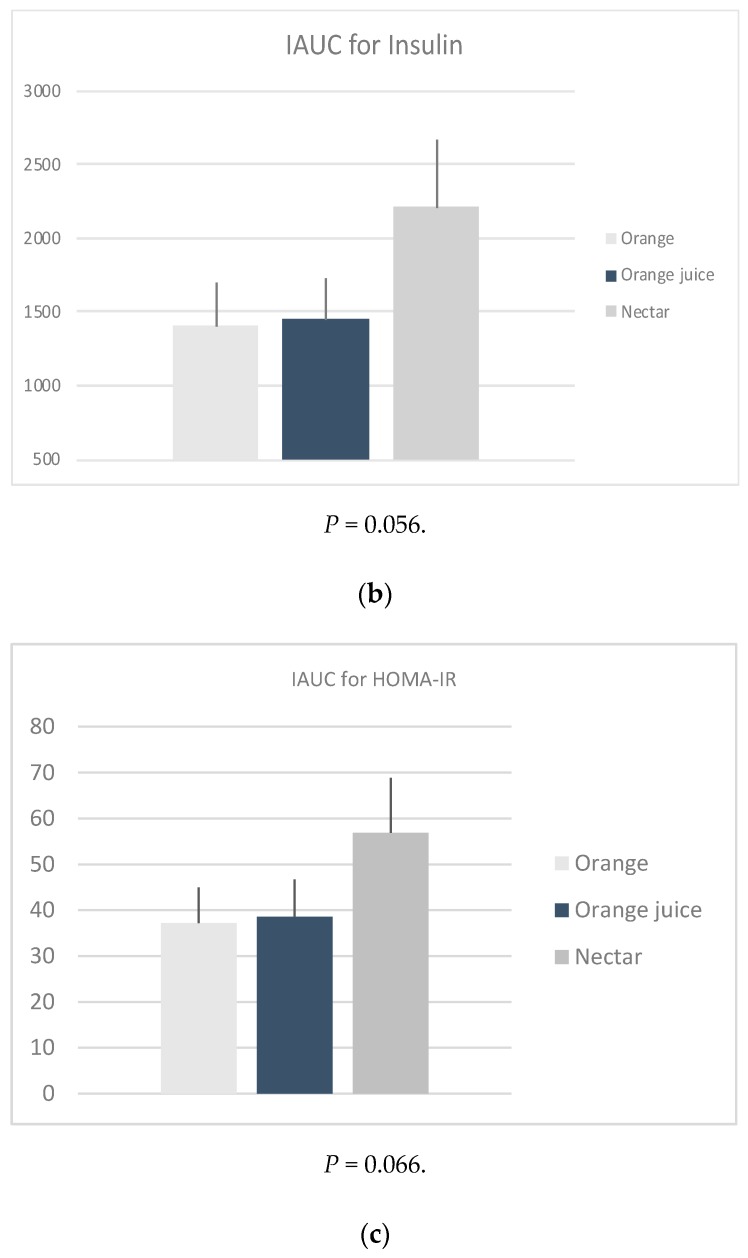
Mean positive incremental area under the curve (iAUC) of the changes in plasma (**a**) glucose, (**b**) insulin and calculated (**c**) HOMA-IR, following four measurements × 30 min of the post intervention test, by sample. * *P*-values derived from mixed effects regression for repeated measures, adjusted for age, weight status, energy, and fiber intake.

iAUC: The glucose (*P* = 0.544), insulin (*P* = 0.056) and HOMA-IR (*P* = 0.066) iAUCs were found to be not significantly different between the three sample meals after adjusting for age, weight, energy, and fiber intake (Figure 1). However, it should be noted that this was possibly due to low sample size.

## 4. Discussion

The results presented here show, and for the first time, the lack of significant acute effect of RO, 100% FOJ, and NSOJ on fasting blood glucose, fasting insulin levels, and HOMA-IR. The analysis also showed that the time × treatment decreasing differences values observed from T120 to T0, were significant for all the three samples with respect to insulin levels and HOMA-IR only.

Currently, diabetes mellitus is one of the most challenging global health epidemics. Cumulative evidence has shown that 100% FJ is positively associated with an increased risk of T2DM in middle-aged Chinese men and women living in Singapore [19] and in middle-aged female Nurses [20]. However, the results of our study are in line with those reported by Wang [16] and by Murphy [17]. Both of them analyzed seven and 18 trials, respectively, and they reported that there was no significant effect of 100% FJs on glycemic control, as measured by fasting blood sugar levels, fasting blood insulin levels, and HOMA-IR. Our findings are also consistent with observational studies showing that consumption of 100% FJ is not associated with an increased risk of T2DM [9]. Recently, a meta-analysis of 15 prospective cohort studies showed that food containing fructose sugars was not associated with an increased risk of T2DM [21]. Another analysis of 11,000 subjects in the European Prospective Investigation into Cancer and Nutrition cohort study showed that there was no association between T2D and the consumption of FJ or NSJ [22]. In summary, most studies suggest that the consumption of FJ is not detrimental to health. 

In this study, the possible reasons contributing to the neutral effects of all three samples on blood glucose and insulin levels may be the high fiber content in whole fruits and the high antioxidant and phytochemical levels in 100% FOJ. Few studies have suggested that the consumption of specific whole fruits (oranges, apples, and berries) is related to a significant reduction in the risk of T2DM risk [7,8]. Other studies have reported that although FJs are deficient in fiber, they contain other important nutritional such as antioxidants and phytochemicals that may prevent T2DM [23].

Our findings, pertaining to NSOJ, do not support the strong associations between sweetened juices and T2DM. In a very recent systematic review from 17 cohorts that included 38,253 cases, it was concluded that habitual consumption of SSBs was positively associated with T2DM even after being adjusted for obesity [24]. Based on the data from another systematic review, which included 310,819 participants, the authors concluded that higher consumption of SSBs was associated with the development of metabolic syndrome and T2DM [25]. SSBs/NSJ consumption has been found to be unfavorable to insulin markers in middle-aged and older adults [26]. Moreover, regular intake of SSBs/NSJ has been found to be associated with a greater increase in insulin resistance and a higher risk of development of prediabetes and CVD [27,28]. The negative results presented in our study may be due to the small sample size, attribute risk bias, or to the fact that all patients had normal blood glucose and insulin levels despite the differences in their body weights.

The lower insulinemic responses observed following the consumption of the three meals were also reflected in the changes in HOMA-IR. Insulin resistance and hyperinsulinemia have been reported as risk factors for CVD [29]. However, our findings indicated that the three meals exert a protective effect at the end of 120 minutes, showing that the consumption of FJs or whole fruits could indeed be beneficial for health.

The findings of this study should be interpreted in the light of several limitations. Although the prospective design of the study was very strong, we cannot omit the fact that the participants have served as their own control. Despite the fact that power calculation showed that the number of participants was adequate, the small size of the study could also be considered as a limitation. Nevertheless, for the first time, we have data available on the effects of whole fruits and FJs on the blood glucose and insulin levels in Emirati women who did not have T2DM.

## 5. Conclusions

RO, FOJ, and NSOJ produced a similar normal glycemic response after adjustment for age, weight, energy, and fiber intake. All three meal samples exerted a favorable effect in terms of eliciting lower insulin levels and HOMA-IR after the 120 minutes. Clinical trials to validate these findings should be conducted in the future but should include more subjects as well as test them for a longer period of time.

## Figures and Tables

**Table 1:** Total sugar content of the three fruit samples.

Fruit Samples	Glucose (g)	Fructose (g)	Sucrose (g)	Maltose (g)	Total (g)
2 Oranges (260 mL)	5.9	6	14 g	0.5	26.4
100% Fruit Juice (265 mL)	7.5	8	11 g	0.5	27
Sweetened Orange Juice (225 mL)	6.5	7	13 g	0.5	27

**Table 2:** Descriptive characteristics of participants.

Variables^1^	Total (*N* = 20)	Normal Weight (*N* = 13)	Overweight & Obese (*N* = 7)	*P*-Value by Weight Status *
Age, years	21.1 (0.9)	21.0 (0.9)	21.3 (0.7)	0.271
Weight (kg)	61.5 (15.3)	53.0 (8.8)	77.2 (11.9)	<0.001
BMI (kg/m^2^)	23.6 (5.4)	20.7 (2.8)	29.1 (2.5)	<0.001
Fasting Glucose ^2^				0.025
RO	85.3 (7.6)	83.5 (6.5)	88.7 (8.8)	
FOJ	86.8 (6.9)	85.6 (7.1)	88.9 (6.2)	
NSOJ	85.6 (6.1)	84.3 (5.5)	88.0 (6.8)	
Fasting Insulin ^2^				0.163
RO	55.5 (20.5)	56.7 (22.6)	53.3 (17.6)	
FOJ	55.7 (27.8)	49.4 (27.2)	67.2 (27.2)	
NSOJ	56.3 (35.2)	50.1 (37.3)	67.7 (30.1)	
Energy (Kcal)	2452.3 (1067.9)	1834.2 (474.5)	3600.3 (832.9)	<0.001
Fiber (g)	14.3 (10.1, 25.4)	11.8 (9.7, 14.5)	28.8 (14.2, 42.6)	<0.001

BMI: Body Mass Index; BP: Blood pressure; * *P*-values based on Student’s *T*-test for continuous binary variables, ANOVA for multiple categories and chi-square test for categorical variables; ^1^ all variables are reported as mean (SD) other than weight status which is reported as percentage (%); ^2^ Total fasting glucose and insulin levels did not differ by treatment (*P* = 0.789 and *P* = 0.763, respectively), only glucose by weight status.

**Table 3:** Mean changes in the metabolic parameters (glucose, insulin, peak glucose, peak glucose as measured in all three samples during 2-h at baseline and post intervention for all three samples (*N* = 20).

	T0	T30	T60	T90	T120	Mean Change	*P*-Value * (Time × Treatment)
	Mean (SD)	Mean (SD)	Mean (SD)	Mean (SD)	Mean (SD)	(T120-T0) Mean (95% CI)	
Serum Glucose							0.462
*RO*	85.3 (7.6)	98.1 (14.9)	86.6 (11.0)	84.6 (6.7)	83.3 (6.7)	−2.0 (−4.94, 0.87)	
*OJ*	86.8 (6.9)	104.0 (14.2)	86.1 (9.3)	84.9 (7.9)	84.7 (7.2)	−2.1 (−4.05, −0.11)	
*SOJ*	85.6 (6.1)	102.3 (17.8)	85.5 (12.4)	83.6 (8.4)	83.3 (7.4)	−2.3 (−4.93, 0.38)	
*P*-value ^+^ (treatment effect)	0.836	0.204	0.649	0.631	0.962		
Serum Insulin **							<0.001
RO	55.5 (20.5)	111.3 (75.4)	60.8 (40.9)	51.4 (31.1)	41.5 (18.4)	−14.0 (−27.05, −0.90)	
*FOJ*	55.7 (27.8)	107.5 (77.2)	60.3 (43.9)	49.2 (26.1)	42.0 (18.8)	−13.7 (−28.80, 1.44)	
*NSOJ*	56.3 (35.2)	136.9 (71.3)	73.8 (56.9)	62.8 (38.6)	47.1 (21.4)	−9.2 (−28.75, 10.30)	
*P*-value ^+^ (treatment effect)	0.925	0.233	0.282	0.123	0.290		
HOMA-IR							<0.001
RO	1.2 (0.5)	2.7 (1.9)	1.4 (1.1)	1.1 (0.8)	0.9 (0.4)	−0.3 (−0.61, −0.03)	
FOJ	1.2 (0.7)	2.8 (2.3)	1.4 (1.1)	1.1 (0.6)	0.9 (0.4)	−0.3 (−0.67, 0.01)	
NSOJ	1.2 (0.8)	3.6 (2.3)	1.7 (1.6)	1.3 (0.9)	1.0 (0.5)	−0.2 (−0.66, 0.22)	
*P*-value^+^ (treatment effect)	0.894	0.163	0.346	0.188	0.283		

All *P*-values derived from mixed effects regression model, for repeated measures, adjusted for age, BMI status, energy intake (kcal) and fiber intake (grams); significance at a *p* = 0.05 level; ^+^ Treatment effect between groups at same time (T120-T0); * Time-treatment interaction effect; ** Regression performed upon log transformation.

## Data Availability

The data used to support the findings of this study are available from the corresponding author upon request.

## Supplementary Materials

The following are available online at https://www.mdpi.com/2072-6643/11/9/2171/s1, Table S1: Mean changes in the metabolic parameters (glucose, insulin) and HOMA-IR levels, as measured in all three samples during 2-h at baseline and post intervention for the three samples, in normal weight participants (*N* = 13), Table S2: Mean changes in the metabolic parameters (glucose, insulin) and HOMA-IR levels, as measured in all three samples during 2-h at baseline and post intervention for all three samples, in overweight/obese participants (*N* = 7).
