# Supplementary material for: Consumption of Raw Orange, 100% Fresh Orange Juice, and Nectar- Sweetened Orange Juice—Effects on Blood Glucose and Insulin Levels on Healthy Subjects
Source: Nutrients. 2019 Sep 10;11(9):2171. doi: 10.3390/nu11092171 (PMC6770506; doi:10.3390/nu11092171)
Supplement: Supplementary file 1 [file nutrients-11-02171-s001.pdf]

Table S1: Mean changes in the metabolic parameters (glucose, insulin) and HOMA-IR levels, as measured in all three samples during 2-h at baseline and post intervention for the 3 samples, in normal weight participants ($N = 13$)

	T0<br>Mean (SD)	T30<br>Mean (SD)	T60<br>Mean (SD)	T90<br>Mean (SD)	T120<br>Mean (SD)	Mean change<br>(T120 - T0)<br>Mean (95% CI)	P-value*<br>(time x treatment)
**Serum Glucose**							0.990
Sample 1	83.5 (6.5)	95.5 (17.6)	84.9 (8.8)	84.5 (5.4)	82.8 (6.1)	-0.7 (-1.90, 0.57)	
Sample 2	85.6 (6.2)	110.2 (11.3)	83.5 (5.8)	84.3 (7.5)	83.1 (6.9)	-2.5 (-3.63, -1.33)	
Sample 3	84.3 (5.5)	99.5 (15.0)	82.0 (10.6)	82.1 (7.6)	82.5 (6.6)	-1.7 (-2.73, -0.77)	
P-value+<br>(treatment effect)	0.650	0.368	0.243	0.259	0.935		
**Serum Insulin****							0.036
Sample 1	58.3 (42.4, 65.3)	109 (33, 166)	52.4 (30.6, 75.7)	45.1 (27, 56.3)	41.7 (32, 46.5)	-14.1 (-30.17, 2.08)	
Sample 2	42.4 (26.0, 59.7)	67.4 (42.4, 103.5)	51.4 (26, 58.3)	44 (35, 56.3)	38.9 (22, 48.6)	-10.8 (-28.41, 6.70)	
Sample 3	47.2 (25.0, 67.4)	107.6 (66, 200.7)	36.8 (29.2, 74.3)	51.4 (30.6, 61.8)	42.4 (33, 61.1)	-3.8 (-29.10, 21.55)	
P-value+<br>(treatment effect)	0.462	0.703	0.923	0.165	0.494		
**HOMA-IR**							<0.001
Sample 1	1.2 (0.5)	2.2 (0.7, 4.4)	0.9 (0.6, 1.7)	0.9 (0.5, 1.2)	0.8 (0.7, 1.0)	-0.3 (-0.64, 0.06)	
Sample 2	1.1 (0.6)	1.7 (0.9, 2.8)	1.0 (0.5, 1.2)	0.9 (0.7, 1.2)	0.8 (0.5, 1.0)	-0.3 (-0.68, 0.11)	
Sample 3	1.0 (0.8)	2.5 (2.0, 5.1)	0.8 (0.6, 1.3)	1.0 (0.7, 1.3)	0.9 (0.7, 1.2)	-0.1 (-0.66, 0.46)	
P-value+<br>(treatment effect)	0.503	0.634	0.786	0.257	0.563		

Sample 1: 2 oranges (260 ml); sample 2: 100% fruit juice (265 ml) ; sample 3: sweetened orange juice (225 ml); All P-values derived from mixed effects regression model, for repeated measures, adjusted for age, weight status, energy intake (kcal) and fiber intake (grams); significance at a=0.05 level; +Treatment effect between groups at same time (T120-T0); *Time-treatment interaction effect; **regression performed upon log transformation

Table S2: Mean changes in the metabolic parameters (glucose, insulin) and HOMA-IR levels, as measured in all three samples during 2-h at baseline and post intervention for all 3 samples, in overweight/obese participants ($N = 7$)

	T0<br>Mean (SD)	T30<br>Mean (SD)	T60<br>Mean (SD)	T90<br>Mean (SD)	T120<br>Mean (SD)	Mean change<br>(T120 - T0)<br>Mean (95% CI)	P-value*<br>(time x treatment)
**Serum Glucose**							0.211
Sample 1	88.7 (8.8)	102.9 (6.7)	89.7 (14.6)	84.7 (9.1)	84.2 (8.1)	-4.6 (-7.02, -2.19)	
Sample 2	88.9 (6.2)	111.2 (16.9)	91.1 (12.9)	85.9 (9.1)	87.6 (7.3)	-1.3 (-2.32, -0.33)	
Sample 3	88.0 (6.8)	108.8 (22.1)	92.1 (13.6)	86.5 (9.8)	84.7 (8.9)	-3.3 (-5.89, -0.62)	
P-value⁺ (treatment effect)	0.764	0.341	0.503	0.612	0.875		
**Serum Insulin****							<0.001
Sample 1	52.1 (43.8, 65.7)	111.8 (65.3, 149.3)	54.9 (31.9, 136.8)	47.9 (39.6, 111.1)	41.7 (32, 46.5)	-13.8 (-43.76, 16.08)	
Sample 2	61.1 (42.4, 79.2)	111.8 (36.8, 229.9)	55.6 (13.9, 120.1)	48.6 (33, 55)	38.9 (22, 48.6)	-18.9 (-55.57, 17.71)	
Sample 3	63.9 (48.0, 95.8)	157.7 (96.5, 188.9)	88 (49.3, 143.1)	71.5 (44, 120.1)	33 (29.2, 80.6)	-19.3 (-58.77, 20.08)	
P-value⁺ (treatment effect)	0.229	0.103	0.109	0.438	0.393		
**HOMA-IR**							<0.001
Sample 1	1.2 (0.4)	2.6 (1.8, 3.8)	1.0 (0.6, 3.6)	0.9 (0.8, 2.6)	0.7 (0.3, 1.0)	-0.4 (-1.07, 0.33)	
Sample 2	1.5 (0.7)	2.6 (1.0, 7.4)	1.2 (0.3, 3.2)	1.0 (0.7, 1.3)	0.9 (0.8, 1.5)	-0.4 (-1.30, 0.43)	
Sample 3	1.5 (0.7)	4.2 (2.3, 4.9)	1.9 (1.1, 3.6)	1.4 (1.0, 2.8)	0.7 (0.6, 1.7)	-0.5 (-1.36, 0.47)	
P-value⁺ (treatment effect)	0.277	0.109	0.136	0.480	0.359		

Sample 1: 2 oranges (260 ml); sample 2: 100% fruit juice (265 ml) ; sample 3: sweetened orange juice (225 ml); All P-values derived from mixed effects regression model, for repeated measures, adjusted for age, weight status, energy intake (kcal) and fiber intake (grams); significance at a=0.05 level; ⁺Treatment effect between groups at same time (T120-T0); *Time-treatment interaction effect; **regression performed upon log transformation.
